# Analysis of the early-flowering mechanisms and generation of T-DNA tagging lines in Kitaake, a model rice cultivar

**DOI:** 10.1093/jxb/ert226

**Published:** 2013-08-21

**Authors:** Song Lim Kim, Minkyung Choi, Ki-Hong Jung, Gynheung An

**Affiliations:** ^1^Crop Biotech Institute, Kyung Hee University, Yongin 446-701, Korea; ^2^Department of Life Sciences, Pohang University of Science and Technology, Pohang 790-784, Korea; ^3^Department of Plant Molecular Systems Biotechnology, Kyung Hee University, Yongin 446-701, Korea

**Keywords:** Flanking sequence database, flowering time genes, GUS, Kitaake, splicing donor and acceptor, T-DNA.

## Abstract

As an extremely early flowering cultivar, rice cultivar Kitaake is a suitable model system for molecular studies. Expression analyses revealed that transcript levels of the flowering repressor *Ghd7* were decreased while those of its downstream genes, *Ehd1*, *Hd3a*, and *RFT1*, were increased. Sequencing the known flowering-regulator genes revealed mutations in *Ghd7* and *OsPRR37* that cause early translation termination and amino acid substitutions, respectively. Genetic analysis of F2 progeny from a cross between cv. Kitaake and cv. Dongjin indicated that those mutations additively contribute to the early-flowering phenotype in cv. Kitaake. Because the short life cycle facilitates genetics research, this study generated 10 000 T-DNA tagging lines and deduced 6758 flanking sequence tags (FSTs), in which 3122 were genic and 3636 were intergenic. Among the genic lines, 367 (11.8%) were inserted into new genes that were not previously tagged. Because the lines were generated by T-DNA that contained the promoterless *GUS* reporter gene, which had an intron with triple splicing donors/acceptors in the right border region, a high efficiency of *GUS* expression was shown in various organs. Sequencing of the GUS-positive lines demonstrated that the third splicing donor and the first splicing acceptor of the vector were extensively used. The FST data have now been released into the public domain for seed distribution and facilitation of rice research.

## Introduction

Rice (*Oryza sativa*. L.) is a model plant for monocot species because its genome has been sequenced (International Rice Genome Sequencing Project, 2005) and it can easily be transformed (Toki *et al.*, 2006; Hiei and Komari, 2008). In addition, insertion mutants have been generated by T-DNA, *Tos17*, *dSpm*, and *Ds* (Krishnan *et al.*, 2009). Their insertion sites have now been determined and the flanking sequence tag (FST) information deposited into public domains such as the Rice Functional Genomic Express Database (RiceGE, http://signal.salk.edu/cgi-bin/RiceGE), Oryzabase (www.shigen.nig.ac.jp/rice/oryzabase/top/top.jsp), and orygeneDB (http://orygenesdb.cirad.fr). The current RiceGE release contains 319,969 insertion sites, including: 106,100 FSTs from POSTECH, Korea (Jeon *et al.*, 2000; Jeong et al., 2002, 2006; An *et al.*, 2003; Ryu *et al.*, 2004); 77,701 from NIAS Tos17 insertion mutants, Japan (Miyao *et al.*, 2003); 47,231 from Rice Mutant Data (RMD), Huazhong Agricultural University, China (Zhang *et al.*, 2006); 28,540 from Eu-CIRAD/Genoplante Oryza Tag Lines (OTL), France (Sallaud *et al.*, 2004); 17,684 sites from UC Davis, USA (Kumar *et al.*, 2005); 11,794 from Taiwan Rice Insertion Mutant, Academia Sinica (Hsing *et al.*, 2007); and 10,281 from SHIP T-DNA, China (Fu *et al.*, 2009).

Insertion mutants are valuable resources, especially when their insertion positions are annotated into a chromosome. This allows one to identify the knockout mutants in a given gene. Systemic phenotyping of the insertion mutants can be used to determine the phenotypic alteration that co-segregates with the insertion element (Krishnan *et al.*, 2009). This reverse-genetics approach has elucidated a number of gene functions (An *et al.*, 2005). Mutants can also be used for verification of functional analysis for a map-based cloned gene (Liu *et al.*, 2009; Piao *et al.*, 2009). Without an insertion mutant allele, one must complement the natural mutant, which requires cloning a full-length gene and transforming the mutant plant. Because transformation efficiency is low in many rice cultivars, such complementation experiments often require great effort and much time.

Some of the T-DNAs used for generating insertion mutants contain a promoterless reporter gene, for example *GUS* or *GFP* (Jeong *et al.*, 2002; Ryu *et al.*, 2004). When T-DNA is inserted within a gene in the same orientation as the mutated gene, a translational fusion can be formed. This allows for easy analysis of temporal and spatial expression patterns associated with the tagged gene (Jefferson, 1989). T-DNA can carry enhancer elements, such as the *35S* enhancer element of cauliflower mosaic virus (Jeong et al., 2002, 2006; Chern *et al.*, 2007; Wan *et al.*, 2009).

When such a T-DNA is inserted near a gene, its expression level can be elevated by the enhancer element. This activation tagging is useful, especially because it generates gain-of-function mutations that are not easily obtained through other mutagenesis procedures. Because gain-of-function phenotypes are dominant, they can be readily distinguished from the somaclonal mutations that may occur during the tissue culture period. Activation tagging saves time and effort when producing overexpression transgenic plants. Therefore, this resource can be used for elucidating gene functions, especially when knockout mutants do not show any phenotypic alteration. This tool can also be employed for identifying agronomically important genes that have been silenced during the domestication of modern cultivars.

Unfortunately, most mutant populations are generated in rice cultivars, such as Nipponbare and Dongjin, that take several months to complete a life cycle. They are difficult to grow to fertility in controlled environments because they require high light intensity and warm temperatures. To overcome this obstacle, it would be desirable for one to be able to create mutant populations in a cultivar with a short life cycle and which grows well in greenhouses and growth chambers year-round. Kitaake is an extremely early-heading rice cultivar originating from Hokkaido, Japan (42–45° N latitude). It flowers 7–8 weeks after sowing, and seeds are harvested in 11–12 weeks.

Early flowering is controlled by several quantitative trait loci (QTLs). These include *Ghd7*/*Hd4*, a major QTL in early-heading cultivars (Nonoue *et al.*, 2008). *Ghd7* encodes a CCT domain-containing protein in the CO-like family (Xue *et al.*, 2008). *Ghd8*/*Hd5* is another allele that exhibits early flowering in those cultivars (Fujino *et al.*, 2013). *Ghd8* encodes an OsHAP3 subunit of the CCAAT-box binding protein (Yan *et al.*, 2010). *OsPRR37* has been identified as the major locus that enhances the photoperiod sensitivity of flowering (Murakami *et al.*, 2005). These traits have been used for generating extremely early flowering cultivars (Shibaya *et al.*, 2011). The current work identified the genes responsible for this phenomenon in cv. Kitaake, generated T-DNA insertion mutants in the cultivar, and examined the efficiency of *GUS* trapping.

## Materials and methods

### Plant materials and growth conditions

Along with the extremely early flowering rice cultivar Kitaake rice, which originated from cv. Hokkaido (42–45° N latitude) and other cultivars (Ichitani *et al.*, 1997; Supplementary Fig. S1, available at *JXB* online), this study also analysed the rice cultivar Dongjin, a mid- to late-flowering cultivar from the southern part of the Korean Peninsula (36–37° N latitude). Plants were grown in the paddy field or in a greenhouse under either short days (12/12 light/dark cycle, 28/25 °C) or long days (14/8 light/dark, 28/25 °C day/night). Light level of the greenhouse was approximately 1000 μmol m^–2^ s^–1^.

### Single-nucleotide polymorphism analyses

A G to T point mutation occurred at 157 nucleotides from the *Ghd7* ATG start codon in cv. Kitaake, generating the *Spe*I (AC*T*AGT) site. To distinguish between Kitaake and Dongjin, this region was amplified with primers Ghd7 SNP F and R, and digested it with *Spe*I (Supplementary Table S1 and Supplementary Fig. S2). Because the point mutations in *OsPRR37* did not generate a restriction enzyme site, the *OsPRR37* single-nucleotide polymorphism was detected by allele-specific PCR (Supplementary Table S1 and Supplementary Fig. S3).

### Generation of T-DNA insertion lines

The binary pGA2715 vector used in this study has been described previously (Jeong *et al.*, 2002). Rice transformation was performed via the *Agrobacterium*-mediated co-cultivation method (Lee *et al.*, 1999). Briefly, *Agrobacterium* strain LBA4404 harbouring pGA2715 was cultured in an AAM medium to an OD_600_ of 0.1–0.2. Scutellum-derived calli were co-cultured with the *Agrobacterium* for 3 d under darkness at 22 °C in a 2N6-AS medium containing 0.2% phytagel. After extensive washing, the calli were cultured for 2 weeks under light at 28 °C on a 2N6D-CH30 medium containing 30mg l^–1^ hygromycin and 250mg l^–1^ cefotaxime. Afterward, they were transferred for an additional 2 weeks of culturing under light at 28 °C in a 2N6-BA medium containing 50mg l^–1^ hygromycin and 250mg l^–1^ cefotaxime. Actively growing calli were then transferred to an MSR16 medium and cultured under light at 28 °C. This resulted in a 70–85% regeneration frequency.

### Isolation of the sequences flanking T-DNA

Genomic DNAs were obtained from leaves of primary transgenic plants as described previously (An *et al.*, 2003). The DNA sequences flanking the inserted T-DNA were determined by the inverse PCR method (An *et al.*, 2003). Briefly, genomic DNA was digested with various restriction enzymes and the cut DNAs were self-ligated with T4 DNA ligase. Nested PCR was conducted to amplify the flanking sequences. The PCR product was directly sequenced with an Applied Biosystems 3730 DNA sequencer. If sequences were not resolved due to multiple amplifications, the amplified bands were separated on an agarose gel and eluted for sequencing.

### Histochemical GUS analyses

GUS staining was performed by a previously described method, with some modifications (Jeon *et al.*, 2000). Plants were cut into approximately 1cm pieces and submerged in staining solution containing 0.5M Na_2_HPO_4_ (pH 7.0), 0.5M NaH_2_PO_4_ (pH 7.0), 0.1% Triton X-100, 0.5M EDTA (pH 8.0), 2% DMSO, 0.1% X-gluc (5-bromo-4-chloro-3-indolyl-β-d-glucuronic acid/cyclohexylammonium salt), 1mM K_3_[Fe(CN)_6_], 1mM K_4_[Fe(CN)_6_].3H_2_O, and 5% methanol. After incubation at 37 °C for 2–24h, the samples were transferred into 70% (w/v) ethanol at 65 °C for more than 1h to remove the chlorophyll. The GUS-stained samples were then soaked in a clearing solution (4M urea, 10% glycerol, and 0.1% triton X-100) for 30–60min at room temperature (Hama *et al.*, 2011). Afterward, the staining pattern was observed with a dissecting microscope (SZX16; Olympus, Tokyo, Japan). For sectioning, tissues were embedded in Technovit 8100 resin (Heraeus Kulzer, Armonk, NY, USA), sectioned to 5-μm thickness, and observed with an optical microscope (BX61; Olympus) using bright- and DIC-field illumination (Jung *et al.*, 2005).

### RNA isolation and quantitative real-time PCR

Total RNA was isolated with Tri Reagent (TaKaRa, Shiga, Japan). First-strand cDNAs were synthesized and utilized for quantitative real-time RT-PCR in a Rotor-Gene 6000 (Corbett Research, Sydney, Australia). Osubi1 served to normalize the cDNA quantity. All experiments were performed at least three times, with three or more samples at each point. Primers sequences for quantitative real-time PCR are shown in Supplementary Table S1. Changes in expression were calculated via the ΔΔCt method. To ensure primer specificity, these experiments were performed when the melting curve showed a single peak. Some PCR products were sequenced to verify the specificity of the reaction.

### Analysis of splicing patterns

For analyses of splicing patterns, genomic DNA was isolated from leaves of the GUS-positive lines, and cDNAs were synthesized from total RNAs of those GUS-positive tissues. The junction regions between the tagged genes and GUS were amplified using the forward primer located in the tagged gene and the reverse primer located in GUS (Supplementary Table S1).

PCR was performed with an initial 5min of denaturation at 95 °C, then 35 cycles of 95 °C for 20 s, 50 or 55 °C for 30 s, and 72 °C for 1min, followed by a final 10min of extension at 72 °C. The PCR products were sequenced by Macrogen (Seoul, Korea).

## Results

### Characteristics of cv. Kitaake rice

**Fig. 1.**
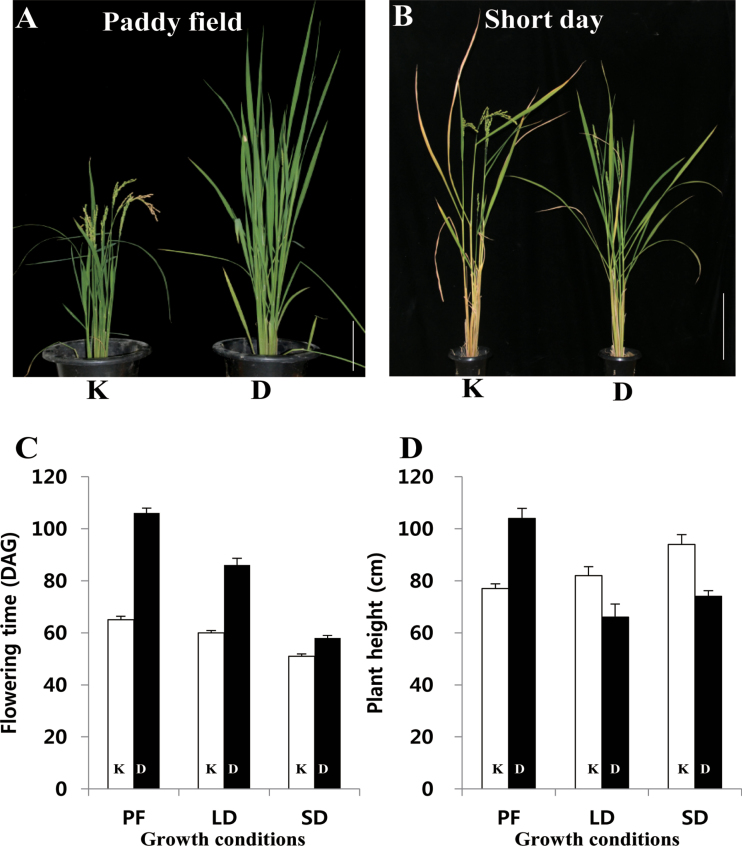
Comparisons between cv. Kitaake and cv. Dongjin plants grown under various conditions. (A, B) Phenotype of cv. Kitaake (K) and Dongjin (D) from paddy field (PF) at 90 DAG (A) or under short day conditions in greenhouse at 100 DAG (B). Bars = 20cm. (C) Flowering time for Kitaake and Dongjin under PF, long days, or short days (*n* = 30) (D) Height of Kitaake and Dongjin under PF, long days, or short days (*n* = 30).

This study compared growth characteristics of cv. Kitaake with those of cv. Dongjin, a cultivar that has previously been used to generate numerous T-DNA insertion lines. In the paddy field (latitude 37° N), Kitaake flowered approximately 65 days after germination (DAG), or 41 d earlier than Dongjin (Fig. 1A, C). To test whether days-to-flowering was affected by photoperiodic conditions, these cultivars were grown under both short and long days. Kitaake flowered earlier under either state: at 51 DAG under short days (7 d earlier than Dongjin) and at 60 DAG under long days (26 d earlier; Fig. 1B, C).

Growth characteristics varied under different environmental conditions. In the paddy, Kitaake plants were about 77cm tall, or 27cm shorter than Dongjin (Fig. 1D). At heading time in the paddy, Kitaake averaged seven tillers per plant versus nine for Dongjin (Supplementary Table S2). However, under short days, Kitaake was as tall as Dongjin (Fig. 1) and had more tillers (Supplementary Table S2). These findings indicated that Kitaake grew better than Dongjin under short day conditions.

**Fig. 2.**
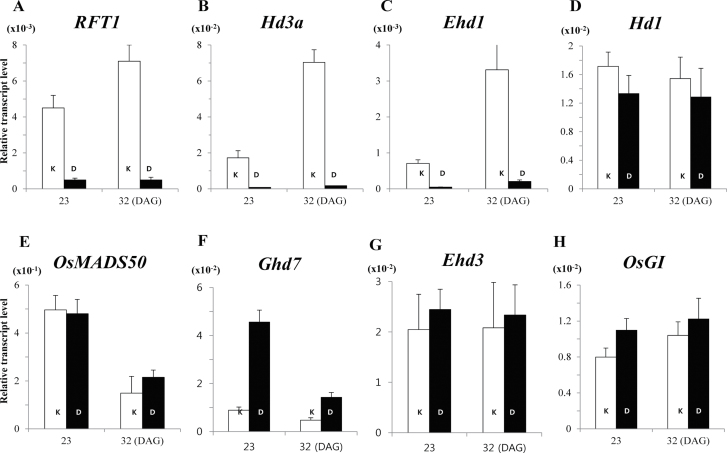
Expression profiles for flowering regulators from cv. Kitaake (K; white) and cv. Dongjin (D; black) at 23 and 32 DAG under long days. RNA was prepared from leaf blades at 2h after turning on lights. Values are mean of two or more independent experiments with standard deviation. Y-axis, relative values between transcript levels for regulatory gene and *Ubi*.

Transcript levels of *RFT1* and *Hd3a*, both florigens in rice, were induced earlier in Kitaake than in Dongjin (Fig. 2A, B). In the latter, these genes were not turned on until 50 DAG when plants were grown under long days. However, they were already expressed at 23 DAG in Kitaake and transcript levels were increased at 32 DAG. Similarly, expression of *Ehd1*, a positive regulator immediately upstream of those florigens, was low in Dongjin whereas expression was detectable at 23 DAG and increased at 32 DAG in Kitaake (Fig. 2C). Because the early flowering phenotype of Kitaake was much stronger under long days or in the paddy, it is likely that a long day-preferential repressor was being suppressed in that cultivar.

Three major regulators—*Hd1*, *OsMADS50*, and *Ghd7*—repress flowering preferentially under long days. For example, *Hd1* enhances flowering time under short days but delays flowering under long days (Yano *et al.*, 2000). *OsMADS50* is an long day-preferential repressor functioning upstream of *Ehd1* (Lee *et al.*, 2004). *Ghd7* is another long day-preferential repressor that has been identified as a major QTL that determines yield (Nonoue *et al.*, 2008; Xue *et al.*, 2008). Whereas the levels of *Hd1* and *OsMADS50* transcripts were not significantly altered in Kitaake (Fig. 2D, E), those of *Ghd7* were much lower in Kitaake than in Dongjin (Fig. 2F).

**Fig. 3.**
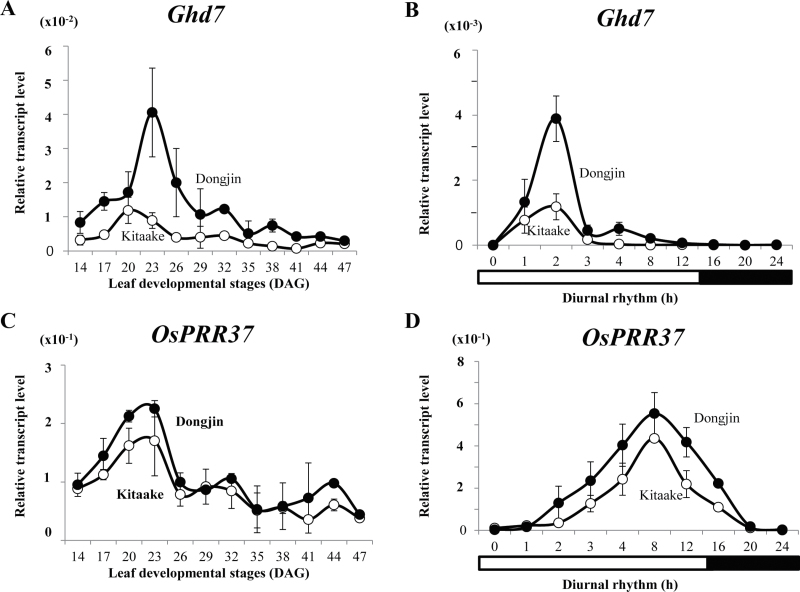
Expression profiles of *Ghd7* and *OsPRR37* in cv. Kitaake (open circles) and cv. Dongjin (closed circles) grown under long days. (A and C) Temporal expression patterns of *Ghd7* and *OsPRR37* in leaf blades from 14 to 47 DAG; samples were prepared at 2h after turning on lights. (B and D) Diurnal rhythm in leaves at 23 DAG. Values are mean of two or more independent experiments and standard deviation. Y-axis, relative values between transcript levels and *Ubi*.

Transcript levels were measured at 3-day intervals, from 14 DAG until 47 DAG. This experiment showed that *Ghd7* transcripts were lower in Kitaake from the early stage of plant development (Fig. 3A). In Kitaake, levels were highest at 2h after the light was turned on and were reduced to low levels thereafter (Fig. 3B). A similar diurnal rhythm was observed in Dongjin, albeit at much higher levels.

Ehd3 is a plant homeodomain (PHD) finger protein that suppresses expression of *Ghd7* (Matsubara *et al.*, 2011). Because *Ghd7* expression was lower in Kitaake, this study examined *Ehd3* transcripts to see whether their levels were higher in Kitaake. However, those levels were similar between the two cultivars (Fig. 2G). The study measured *OsGI*, another repressor of *Ghd7* (Hayama *et al.*, 2003; Itoh *et al.*, 2010), and found little difference in its transcript levels (Fig. 2H). Also, there were no alterations in levels of *OsPRR37*, *OsPhyA*, *B*, or *C* (Fig. 3C, D and Supplementary Fig. S4). Therefore, their expression levels were not the primary reason for this reduction in *Ghd7* transcripts in Kitaake.

To determine whether alterations in protein sequences might cause a decline in *Ghd7* expression and delay early flowering, genomic DNA of the flowering-time regulatory genes from Kitaake were sequenced and compared them with those from cv. Nipponbare. No changes were detected in the genic region or the 2- to 2.5-kb promoter area of *OsPhyA*, *OsPhyC*, *Se5*, *Ehd3*, *ELF3*, *OsGI*, or *Hd5/Ghd8*. Along with synonymous missense mutations in the coding regions for *OsPhyB* (A to G, 1986bp from ATG) and *OsMADS50* (G to T, 157bp from ATG), an amino acid change from histidine (*C*AT) to tyrosine (*T*AC) was found at the 106th residue in *Hd1*. *Ghd7* has a single-nucleotide polymorphism, altering the 53rd amino acid glutamate (*G*AG) to the stop codon (*T*AG) in Kitaake, and generating a non-functional protein due to early termination (Supplementary Fig. S2). In *OsPRR37*, five mutations occurred: four point mutations and one insertion (Supplementary Fig. S3). Three of these point mutations caused changes in the following amino acid residues: the 47th arginine (C*G*G) to proline (C*C*G), 222nd aspartic acid (*G*AT) to asparagine (*A*AT), and 710th leucine (C*T*G) to proline (C*C*G). One of the point mutations and a 2-bp insertion occurred in the 5th intron. Therefore, it appeared that the early flowering phenotype in Kitaake is primarily due to those mutations in *Ghd7* and *OsPRR37*. *Hd1* was excluded because Dongjin also had the same point mutation in that gene. In addition, knocking out *Hd1* should have caused late flowering under short days and early flowering under long days. Instead, Kitaake flowered earlier under both photoperiods.

### Roles of *OsPRR37* and *Ghd7* mutations in controlling flowering time

**Fig. 4.**
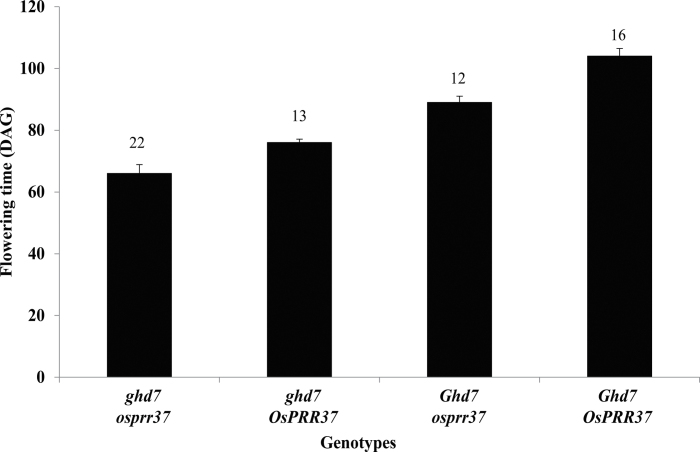
Analysis of flowering time in F2 population for cross between cv. Kitaake and cv. Dongjin. *Ghd7* and *OsPRR37* are Dongjin alleles and *ghd7* and *osprr37* are Kitaake alleles. A total of 124 F2 progeny were analysed. Numerals above the bars indicate the number of double homozygotes in each genotype class.

The effects of the Kitaake *OsPRR37* and *Ghd7* alleles were investigated by crossing Kitaake and Dongjin. From the F1 hybrid, 124 F2 progeny were grown in the paddy field and genotyped according to those alleles. The progeny with Kitaake *OsPRR37*and *Ghd7* alleles flowered at 66±2.9 DAG, similar to the Kitaake parent (Fig. 4). By comparison, the progeny with Dongjin *OsPRR37*and *Ghd7* alleles flowered at 104±2.5 DAG, similar to the Dongjin parent. This indicated that the two alleles are major contributors to early flowering in Kitaake. When the progeny carried the defective *ghd7* from Kitaake and the normal *OsPRR37* from Dongjin, they flowered at 76±1.1 DAG), or 28 d earlier than the progeny with the Dongjin alleles. This demonstrated that the mutation in *Ghd7* causes early flowering in Kitaake. Finally, when the progeny carried the defective *osprr37* from Kitaake and the normal *Ghd7* allele from Dongjin, they flowered at 89±2.0 DAG, or 15 d earlier that those with the Dongjin *OsPRR37* allele, thereby supporting the conclusion that the mutations in *OsPRR37* also contribute to early flowering in Kitaake.

### Generation of T-DNA tagging lines in Kitaake

This study group has previously generated T-DNA tagging lines in cultivars Dongjin and Hwayoung, both of which take approximately 100–110 d to flower in the paddy field (Jeong et al., 2002, 2006). Neither grows well in the greenhouse during the cold season. The current work produced 10 000 primary transformants in Kitaake and grew them to maturity in the greenhouse. Seeds were harvested from approximately 6000 plants. To find the insertion position, inverse PCR was used to determine the sequences flanking the T-DNA. This resulted in the identification of 6758 FSTs from 4614 lines. The FSTs were located on rice chromosomes via a blastn homology search. Among the 6758 inserts, 3122 (46.2%) were genic. The 300-bp UTR from ATG and the stop codons were considered to be genic regions. T-DNAs were located at the 5′-UTR (548 lines), exons (1138 lines), introns (1062 lines), and 3′-UTR (374 lines). Among the 3122 genic inserts, T-DNA was inserted into the gene that was not previously tagged by T-DNA or transposons in 367 lines (11.8%). These FST data have now been released into the public domain RiceGE Database (http://signal.salk.edu/cgi-bin/RiceGE) for purposes of seed distribution (Table 1).

**Table 1.** Description of T-DNA insertions in cv. Kitaake T-DNA tagging lines

Location of T-DNA insertion	No. of inserts	No. of genes
Genic	3122	2666
5′-UTR (300bp upstream)	548	475
Exon	1138	958
Intron	1062	910
3′-UTR (300bp downstream)	374	323
Intergenic	3636	3009
Total	6758	5675

**Fig. 5.**
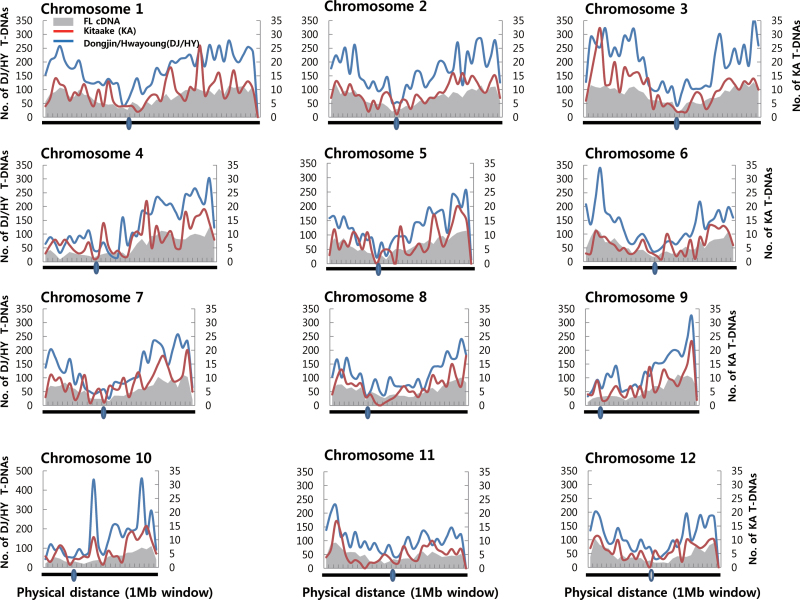
Distribution of T-DNA insertions and KOME full-length cDNA along rice chromosomes. Red = Kitaake T-DNA insertions; blue = Dongjin/Hwayoung T-DNA insertions; grey = KOME full-length cDNA. Grey circles indicate the centromeric regions.

T-DNAs were evenly distributed among 12 chromosomes, proportional to the chromosome size (Table 2). The highest frequency was at chromosome 1, which is the largest in the genome. Distribution of those insertions over the 12 chromosomes was very similar in Kitaake to those for Dongjin and Hwayoung (Fig. 5). The frequency of insertion was lower in the heterochromatin regions near the centromere and higher in the distal euchromatin regions. Similar results were obtained when the current findings were compared with those from T-DNA lines recorded at RMD and OTL (Supplementary Fig. S5).

**Table 2.** Distribution of T-DNA insertions in cv. Kitaake T-DNA tagging lines

Chromosome	Size, Mb (%)	Pseudomolecule, *n* (%)	Full-length cDNA, *n*(%)	Genic insertion, *n* (%)	Intergenic insertion, *n* (%)
1	43.3 (11.6)	6542 (11.7)	4026 (14.1)	421 (13.5)	450 (12.4)
2	35.9 (9.6)	5387 (9.6)	3196 (11.2)	336 (10.8)	452 (12.4)
3	36.4 (9.8)	5573 (9.9)	3569 (12.5)	405 (13.0)	520 (14.3)
4	35.5 (9.5)	5322 (9.5)	2531 (8.9)	305 (9.8)	356 (9.8)
5	30.0 (8.0)	4579 (8.2)	2313 (8.1)	245 (7.8)	302 (8.3)
6	31.2 (8.4)	4724 (8.4)	2292 (8.0)	218 (7.0)	237 (6.5)
7	29.7 (8.0)	4462 (8.0)	2183 (7.6)	275 (8.8)	264 (7.3)
8	28.4 (7.6)	4194 (7.5)	1933 (6.8)	198 (6.3)	245 (6.7)
9	23.0 (6.2)	3408 (6.1)	1605 (5.6)	183 (5.9)	226 (6.2)
10	23.2 (6.2)	3517 (6.3)	1538 (5.4)	159 (5.1)	178 (4.9)
11	29.0 (7.8)	4166 (7.4)	1685 (5.9)	171 (5.5)	204 (5.6)
12	27.5 (7.4)	4022 (7.2)	1693 (5.9)	206 (6.6)	202 (5.6)
Total	373.2 (100)	56,081 (100)	28,564 (100)	3122 (100)	3636 (100)

### Gene trapping using the *GUS* reporter gene

The pGA2715 vector used here carries the promoterless *GUS* reporter gene, with an intron containing triple splicing donors/acceptors immediately next to the right border (RB; Supplementary Fig. S6). Therefore, some of the tagging lines were capable of generating a translational fusion between the tagged gene and *GUS*. Among the T-DNA tags found in the genic regions, the orientation of *GUS* was identical to that of the tagged gene in 1366 inserts: 267 in the 5′-UTR, 561 in the exons, and 538 in the introns. Inserts in the 3′-UTR were excluded because they did not form a translational fusion.

This work assayed 1213 lines for *GUS* activity. The remaining 153 were not evaluated because they produced too few seeds. A total of 159 lines (13.4%) proved GUS-positive; 118 showed staining in the roots, 102 in the shoots, and 88 in germinating seeds. Some of these (63 lines) indicated preferential expression in only one of those plant parts, whereas 53 lines showed *GUS* staining in all three (Supplementary Table S3).

**Fig. 6.**
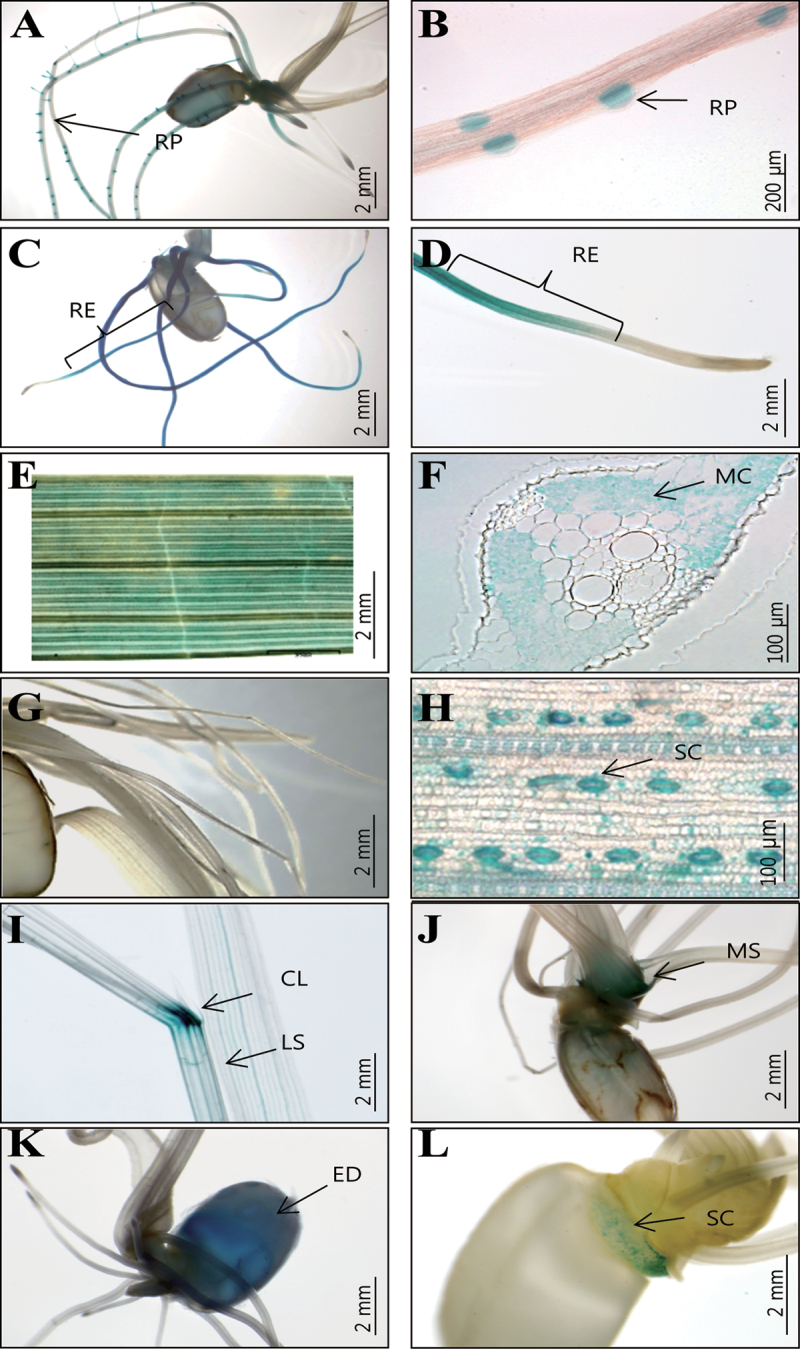
Patterns of *GUS* expression in 10 DAG seedlings. (A, B) Preferential expression in root primordium (RP) from line K-00491. (C, D) Preferential expression in root elongation zone (RE) from line K-01188. (E, F) Mesophyll cell (MC)-specific expression from line K-04449. (G, H) Stomata cell (SC)-preferential expression from line K-00221. (I) *GUS* expression in collar region (CL) from line K-00157; LS = leaf sheath. (J) *GUS* expression in mesocotyl region (MS) from line K-04010. (K) *GUS*-staining in endosperm (ED) of germinating seeds from line K-04210. (L) Preferential expression in scutellum (S) of germinating seeds from line K-04980. Bars = 2mm (A, C–E, G, I–L), 200 μm (B), and 100 μm (F, H).

A majority of the positive lines (80) with staining in the roots showed a *GUS* expression pattern ubiquitous in all root organs while 61 also exhibited expression in the shoots or germinating seeds. Some of the remaining lines had staining in a specific region within the roots while others were GUS-positive in two or more organs (Supplementary Table S4). For example, *GUS* was preferentially expressed in the root primodium in line K-00491 (Fig. 6A, B). T-DNA was tagged in Os09g21000, encoding a putative potassium transporter. Preferential staining was observed in the elongation zone of line K-01188 (Fig. 6C, D). T-DNA was also found in Os05g49300, encoding an iron-sulphur cluster assembly enzyme, or ISCU.

Among the 102 positive lines in the shoots, 26 showed *GUS* expression in all organs, including the leaf blade, sheath, coleoptile, mesocotyl, and collar (laminar joint). Of these lines, 24 also had expression in the roots and germinating seeds. Some of the remaining lines displayed staining in only a specific region while others were GUS-positive in two or more shoot organs (Supplementary Table S4).

Mesophyll cell-specific *GUS* expression was observed in line K-04449, where T-DNA was inserted in Os02g13360 (Fig. 6E, F). In line K-00221, *GUS* was strongly expressed in the stomata (Fig. 6G, H). T-DNA was also located in Os08g35190, which encodes a putative auxin-repressed protein. In line K-00157, expression was strong in the collar region. T-DNA was inserted in Os07g05190, which encodes a leucine-rich repeat (LRR) family protein (Fig. 6I). In line K-04010, expression was preferential in the mesocotyl region (Fig. 6J).

*GUS* expression was also observed in various tissues of germinating seeds (e.g. the aleurone layer, scutellum, and endosperm; Supplementary Table S3). Endosperm-preferential expression was found in five lines. For example, staining was noted in line K-04210, where T-DNA was inserted into Os02g13360 (Fig. 6K). This expression pattern coincided with that from public array data (www.ricearray.org; Jung *et al.*, 2011). Finally, in line K-04980, *GUS* expression was observed in the scutellum (Fig. 6L), where T-DNA was inserted into Os12g38880, which encodes a pentratricopeptide-like helical protein.

**Fig. 7.**
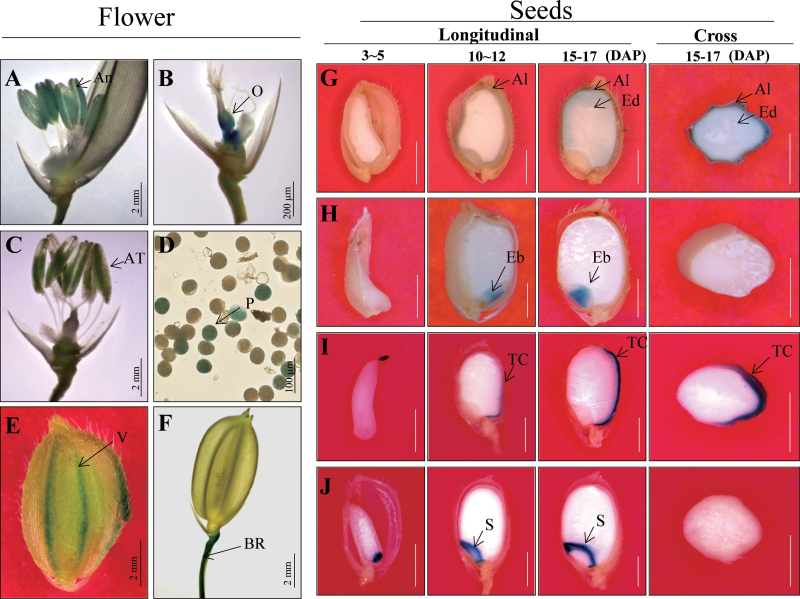
Patterns of *GUS* expression in reproductive organs. Mature florets (A–F) and developing seeds (G–J) at three developmental stages (3–5, 10–12, and 15–17 days after pollination) were examined. (A) Anther (An) from line K-00143. (B) Ovary (O) from line K-00188. (C, D) Pollen (P) from line K-00241. (E) Vascular bundle (V) of palea/lemma from line K-01531. (F) Branch rachis (BR) from line K-00561. (G) Aleurone layer (Al) from line K-04584. Ed, endosperm. (H) Plumule of embryo (Eb) from line K-03829. (I) Endosperm transfer cell (TC) at dorsal region of endosperm from line K-00350. (J) Scutellum (S) from line K-01208. Bars = 2mm (A, C, E–J), 200 μm (B), and 100 μm (D).

This study also assayed 421 lines at various reproductive stages. Staining was found in the mature floral organs and developing seeds of 80 lines (19.0%; Supplementary Table S5). Preferential expression was observed in the anther (line K-00143), ovary (line K-00188), pollen (line K-00241), vascular bundle in the palea/lemma (line K-01531), and branch rachis (line K-00561; Fig. 7A–F). Details for these patterns of expression are presented in Supplementary Table S6. Seeds were examined during three periods of development: 3–5, 10–12, and 15–17 days after pollination. In some lines, such as line K-04584, *GUS* expression was detected preferentially in the aleurone layer (Fig. 7G). In K-03829, expression was preferential to the plumule in the embryo region (Fig. 7H). For K-00350, *GUS* was detected in the dorsal region of the endosperm (Fig. 7I), while expression was preferential to the scutellum in K-01208 (Fig. 7J).

The frequency of occurrence for GUS-positive lines was slightly higher in exons (21.4%, 108/505) than in either introns (15.7%, 75/478) or the 5′-UTR (13.0%, 30/230).

### Splicing patterns of GUS-positive lines

Because the pGA2715 vector includes an intron with triple splicing donors/acceptors immediately next to the RB, this feature allows for a translational fusion to occur between the tagged gene and *GUS* at high frequency if all three splicing donors and acceptors are utilized. However, the frequency of GUS-positive lines was much lower here than expected. Therefore, this work investigated splicing patterns in the lines showing *GUS* expression. Genomic DNA and cDNA were prepared, and the junction regions between the tagged gene and *GUS* were determined.

**Fig. 8.**
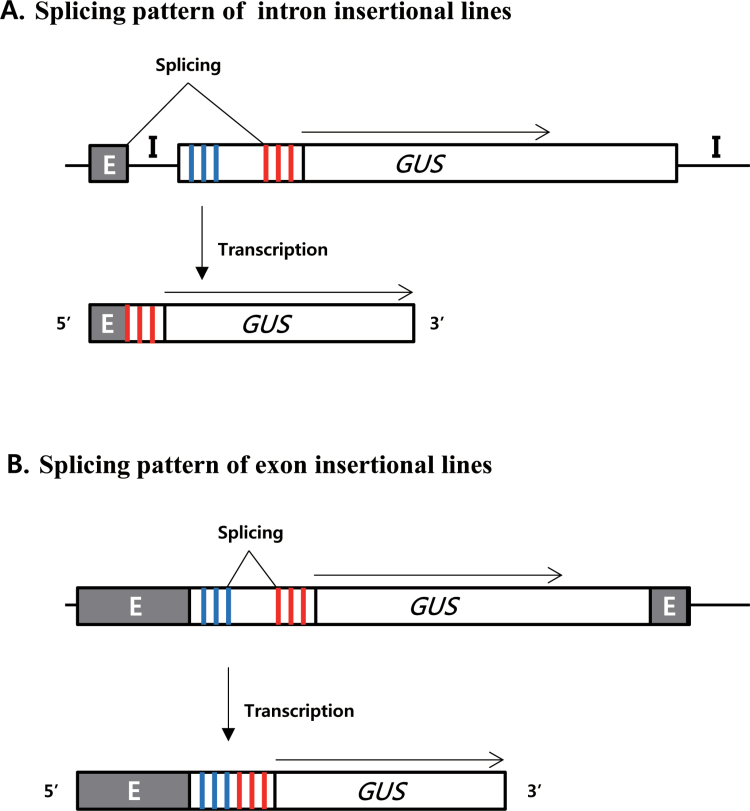
Splicing patterns. (A) Intron insertional lines. (B) Exon insertional lines. Upper, schematic diagrams of genomic DNA at T-DNA insertion sites; lower, fusion transcripts between tagged genes and *GUS*. Blue = splicing donors; red = splicing acceptors; E = exons; I = introns.

When T-DNA was inserted into an intron, the splicing donor from the tagged gene and the acceptor from the vector were used to generate fusion transcripts. If all three acceptors were employed, three different transcripts would be created but only one would make a proper reading frame, thereby forming a translational fusion. GUS-positive lines where T-DNA was inserted into an intron were selected. Sequencing revealed that these plants produced only one type of fusion transcript. For example, in line K-01715, T-DNA was inserted into intron 1. Analysis of the fusion transcript between the tagged gene and *GUS* revealed only one type of transcript, which utilized the first splicing acceptor (Fig. 8A). Similar splicing patterns were observed from line K-04652, where T-DNA was inserted into intron 1 of Os05g49180 (Table 3). Therefore, this indicated that the first splicing acceptor of the intron located in front of *GUS* was dominantly utilized.

**Table 3.** Splicing patterns for cv. Kitaake GUS-positive linesAll lines were generated using splicing acceptor 1.

Line	Locus	Putative function	Position	Splicing donor
K-01715	Os06g40620	SNF7 domain-containing protein	Intron 1	Tagged intron
K-04652	Os05g49180	DUF1296 domain-containing protein	Intron 1	Tagged intron
K-00313	Os04g57880	Heat shock protein DnaJ	Exon 9	3
K-03357	Os02g58460	β-Catenin-like protein 1	Exon 8	3
K-04431	Os07g06300	Ethylene-insensitive protein 2	Exon 8	3
K-01785	Os01g66940	Kinase, pfkB family	Exon 4	3
K-01651	Os01g15020	Lissencephaly type-1-like homology motif	Exon 24	3
K-05933	Os07g14850	CESA6–cellulose synthase	Exon 12	3
K-00181	Os10g33940	Auxin response factor 18	Exon 1	3
K-03285	Os02g46480	Unknown	Exon 1	3
K-00356	Os03g14170	3-Ketoacyl-CoA synthase	Exon 1	3
K-03673	Os03g06710	PPR domain-containing protein	Exon 1	3
K-03284	Os02g46480	Hypothetical protein	5′-UTR	3

When T-DNA was inserted into an exon, the intron located in front of *GUS* would have been spliced. Because this study had three splicing donors and three splicing acceptors, nine different, alternative splicing products could have been generated if all were utilized. However, when sequenced here, only one type of transcript was observed. For example, in line K-04431 where T-DNA was inserted into exon 8 of Os07g06300 (encoding an ethylene-insensitive protein), the third splicing donor and the first splicing acceptor were used to make the mature mRNA. This splicing allowed for a translational fusion between the ethylene-insensitive protein and *GUS* (Fig. 8B). Like line K-04431, nine additional lines has T-DNA inserted into an exon (Table 3). This same splicing pattern was observed when T-DNA was inserted into the 5′-UTR (Table 3). All of these findings demonstrated that only one each of the splicing donors and acceptors was predominantly used, regardless of the insertion positions.

## Discussion

The availability of in-depth genome sequences and abundant public genetics resources make rice a good model system for understanding plant gene functions. However, because most cultivars do not perform well in controlled environments, it would be desirable to use one that grows rapidly in greenhouses and growth chambers year-round. This study proposed Kitaake as its model. It flowers about 1 month earlier than most japonica cultivars. Because it requires less light for optimal growth and is more cold tolerant than other rice cultivars, it grows much better in greenhouses even during the cold season.

Sequencing the flowering-control genes in Kitaake revealed that the amino acid sequences of *Ghd7* and *OsPRR37* were altered when compared with Dongjin. A point mutation in the former caused early termination of Ghd7 translation, resulting in a complete loss of function. As expected, transcript levels for *Ehd1*, which is immediately downstream of *Ghd7*, were elevated in Kitaake. *Ehd1* merges various environmental and developmental signals and controls the expression levels of *Hd3a* and *RFT1*, both florigens in rice (Komiya *et al.*, 2008). This same point mutation has been found in several other early-heading cultivars, such as Hayamasari, Mudandian8, and Hejiang19 (Xue *et al.*, 2008; Matsubara *et al.*, 2011). Therefore, it appears that the mutant allele is common to early-flowering cultivars. In *OsPRR37*, three mutations cause amino acid substitutions. The cultivar Kasalath also has a severe lesion in that gene, leading to premature termination (Murakami *et al.* 2005).

To evaluate the roles of the *Ghd7* and *OsPRR37* alleles in Kitaake, this study generated F2 progeny from the cross between Kitaake and Dongjin. Analyses showed that the plants with Kitaake *OsPRR37* flowered 15 d earlier that those with Dongjin *OsPRR37*, whereas Kitaake *Ghd7* accelerated flowering by 28 d. This experiment indicated that both the *Ghd7* and *OsPRR37* alleles from Kitaake are linked to early flowering and that the effects are more severe with *Ghd7* than with *OsPRR37*.

Asian rice can be classified into five types (*Ghd7*-0, -0a, -1, -2, and -3),according to their variations in *Ghd7* transcripts (Xue *et al.*, 2008). Early-flowering cultivars such as Kitaake belong to type *Ghd7*-0a, carrying a non-functional allele. Transforming members of this type with the functional *Ghd7* allele delays flowering time significantly. In cultivars with the non-functional allele, expression of *Ehd1* is greatly enhanced, suggesting that *Ghd7* functions upstream of *Ehd1* as a repressor.

The OsPRR37 protein is highly homologous to *Arabidopsis* PRR proteins, which operate within a transcriptional feedback loop of clock-associated genes. For example, the *Arabidopsis* PRR proteins act as transcriptional repressors of *Circadian Clock-Associated 1* (*CCA1*) and *Late Elongation Hypocotyl* (*LHY*), which are the central oscillators of the circadian clock in plants (Nakamichi *et al.* 2010). In *Arabidopsis*, CCA1 and LHY control flowering time through a genetic pathway that appears to be independent of floral regulators GIGANTEA and CONSTANS (Fujiwara *et al.* 2008). They repress expression of *Short Vegetative Phase* (*SVP*), encoding a MADS box transcription factor that inhibits FT expression (Fujiwara *et al.*, 2008). Therefore, it could be speculated that OsPRR37 also acts as a repressor of rice *CCA1* and *LHY*, which control a flowering-time regulator. In Kitaake, expression of *Ghd7* was reduced, possibly due to mutations in *OsPRR37*. However, analysis of T-DNA insertion mutants suggested that *OsPRR37* controls *Hd3a* independently from *Ehd1*, *Hd1*, and *Ghd7* (Koo *et al.*, 2013). Reduced *Ghd7* transcript levels in Kitaake might be caused by nonsense-mediated mRNA decay. Further analysis is needed to elucidate the *OsPRR37*-mediated genetic pathway.

Annually, this research group distributes more than 3000 T-DNA insertion lines generated in Dongjin and Hwayoung. However, many laboratories experience difficulty in growing those plants to maturity. Due to space limitations in the GMO-certified paddy field, this group is unable to satisfy the demand for amplifying seeds for the scientific community. Therefore, T-DNA insertional mutant populations in Kitaake were generated instead. The transformation efficiency of cultivar Kitaake is comparable to that of Dongjin, a cultivar that historically transforms well. As a first step toward producing a larger population, approximately 10 000 insertion lines have now been generated and the positions of 6758 insertion sites have been determined. As expected, their patterns of T-DNA distribution on the chromosomes are similar to those previously reported (Sallaud *et al.*, 2004; Jeong *et al.*, 2006). The frequency of knocking out a gene is approximately 46%, which is also close to that achieved in earlier experiments (An *et al.*, 2003; Jeong *et al.*, 2006).

Because the tagging lines carry the promoterless *GUS* within the T-DNA, some are GUS-positive, including 9.7% of the lines in their roots, 9.0% in the shoots, and 7.3% in germinating seeds. In reproductive organs, 23.5% of the lines are GUS-positive. Those showing organ-specific expression should be good resources for further study. For example, laminar joint-preferential *GUS* expression occurs in line K-00157, where T-DNA is inserted into Os07g05190, which encodes a putative LRR family protein. The laminar joint is a mechanical tissue that bends the leaf blade away from the vertical axis toward the abaxial side when the leaf blade and leaf sheath are fully elongated (Hoshikawa, 1989). This laminar joint angle normally increases in parallel with the concentration of brassinosteroid, but that angle is narrower in *brassinosteroid insensitive 1* (*OsBRI1*) mutants than in the wild type (Yamamuro *et al.*, 2000; Jeong *et al.*, 2012). Because OsBRI1 (Os01g52050) also has an LRR domain, these two proteins may share a similar molecular mechanism. Therefore, this line would be a good resource for studying the regulation of laminar joint movement in relation to brassinosteroids.

As another example, line K-04980 shows scutellum-preferential *GUS* expression. T-DNA interrupts Os12g38880, which encodes a putative pentratricopeptide-like helical protein. Such proteins are involved in gibberellin, cytokinin, and auxin responses, as well as ethylene biosynthesis (Schapire *et al.*, 2006). In *Arabidopsis*, mutations within pentatricopeptide repeat (PPR) genes, including *EMB 175*, *CYK8*, and *DYH216*, have a phenotype of embryo defects (Lurin *et al.*, 2004; Cushing *et al.*, 2005). Therefore, this particular rice line would be useful in studying embryo development.

The T-DNA vector used in this study contains an intron with multiple splicing donor and acceptor sequences, allowing for high-efficiency *GUS*-tagging. However, the efficiency of *GUS* trapping has been much lower than expected. This indicates that not all of the splicing sequences are utilized in the plants. Sequencing of the GUS-positive lines revealed that only the third donor and the first acceptor are efficiently used to process pre-mRNA. Therefore, two-thirds of the T-DNA inserts cannot make a translational fusion. However, because those T-DNA insertion sites are known and the splicing patterns can be predicted, it is possible to deduce the lines that are capable of generating a functional translation fusion between the tagged gene and *GUS*.

Although public databases contain more than 300000 FSTs, approximately 40% of the rice genes have not yet been mutated. This work has described 367 T-DNA tags in new genes that were not previously reported and 2755 tags to genes tagged earlier, thereby providing additional alleles. Thus, generation of additional mutant lines in Kitaake will facilitate functional analyses of rice genes.

## Supplementary material

Supplementary data are available at *JXB* online.

Supplementary Fig. S1. Pedigree of cv. Kitaake, with tree drawn based on information from Ichitani *et al.* (1997).

Supplementary Fig. S2. Schematic diagram of *Ghd7* and GHD7 protein from cv. Kitaake and cv. Dongjin.

Supplementary Fig. S3. Schematic diagram of *OsPRR37* and its coding protein from cv. Kitaake and cv. Dongjin.

Supplementary Fig. S4. Expression profiles of *OsPRR37*, *OsPhyA*, *OsPhyB*, and *OsPyhC* in cv. Kitaake (K) and cv. Dongjin (D) at 23 and 32 DAG under long days.

Supplementary Fig. S5. Distribution of T-DNA insertions from cv. Kitaake, RMD, and OTL along rice chromosomes.

Supplementary Fig. S6. Schematic diagram of T-DNA in pGA2715 vector, and DNA sequence in RB region.

Supplementary Table S1. Primer sequences used in this study.

Supplementary Table S2. Comparison of cv. Kitaake and cv. Dongjin plants grown in paddy field or under short-day conditions.

Supplementary Table S3. Frequencies of *GUS* expression in 10 DAG seedlings, based on 1213 lines.

Supplementary Table S4. Patterns of *GUS* expression according to tagging lines in 10 DAG roots, shoots, and germinating seeds.

Supplementary Table S5. Frequencies of *GUS* expression at various reproductive stages, based on 421 lines.

Supplementary Table S6. Patterns of *GUS* expression in reproductive organs.

Supplementary Data
