# Supplementary material for: Analysis of the early-flowering mechanisms and generation of T-DNA tagging lines in Kitaake, a model rice cultivar
Source: J Exp Bot. 2013 Aug 21;64(14):4169–82. doi: 10.1093/jxb/ert226 (PMC3808308; doi:10.1093/jxb/ert226)
Supplement: Supplementary Data [file supp_64_14_4169__index.html]

Analysis of the early-flowering mechanisms and generation of T-DNA tagging lines in Kitaake, a model rice cultivar — Analysis of the early-flowering mechanisms and generation of T-DNA tagging lines in Kitaake, a model rice cultivar — Supplementary Data 

# Analysis of the early-flowering mechanisms and generation of T-DNA tagging lines in Kitaake, a model rice cultivar

## 

Data files

**Files in this Data Supplement:**

- Supplementary Data - Supplementary Data
- Supplementary Data - Supplementary Data
