# Supplementary material for: Analysis of the early-flowering mechanisms and generation of T-DNA tagging lines in Kitaake, a model rice cultivar
Source: J Exp Bot. 2013 Aug 21;64(14):4169–82. doi: 10.1093/jxb/ert226 (PMC3808308; doi:10.1093/jxb/ert226)
Supplement: Supplementary Data [file supp_ert226_jexbot102269_file001.pdf]

JEXBOT/2012/089920

Analysis of the early-flowering mechanisms and generation of T-DNA tagging lines in 'Kitaake', a model rice cultivar

Song Lim Kim[1,2], Minkyung Choi[1,3], Ki-Hong Jung[1,3], and Gynheung An[1,3*].

[1]Crop Biotech Institute, Kyung Hee University, Yongin 446-701, Korea
[2]Department of Life Sciences, Pohang University of Science and Technology, Pohang 790-784, Korea
[3]Department of Plant Molecular Systems Biotechnology, Kyung Hee University, Yongin 446-701, Korea

Running title: T-DNA tagging lines in 'Kitaake'

* To whom correspondence should be addressed: Email: genean@khu.ac.kr

Supplementary Figures

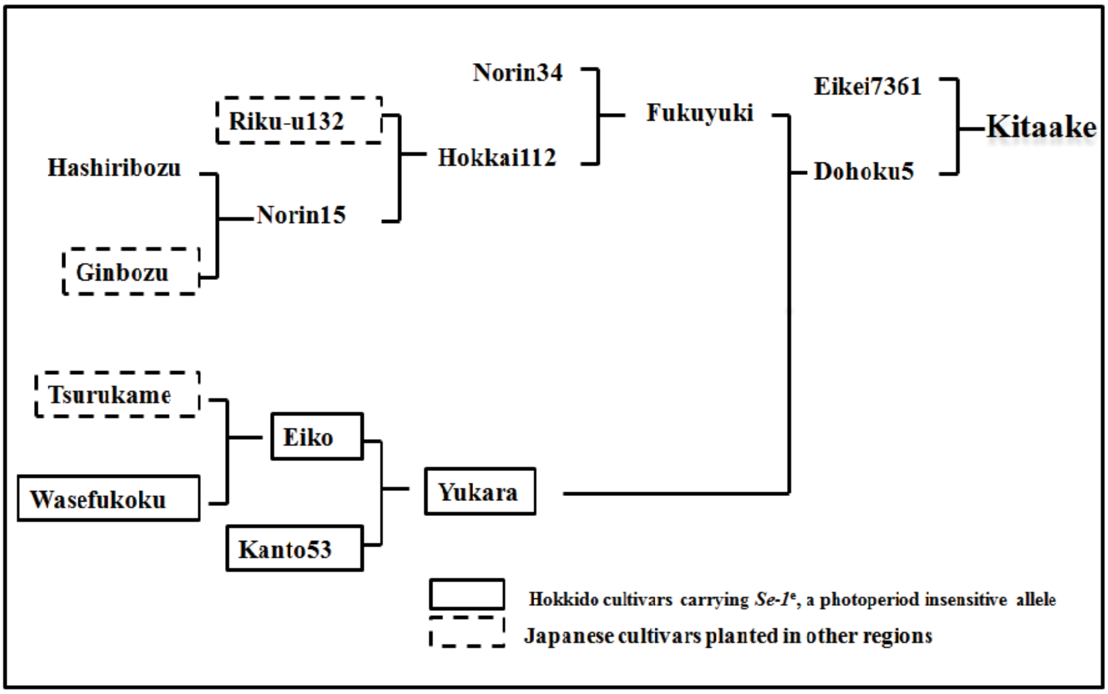

Figure S1. Pedigree of 'Kitaake', with tree drawn based on information from Ichitani *et al*. (1997).

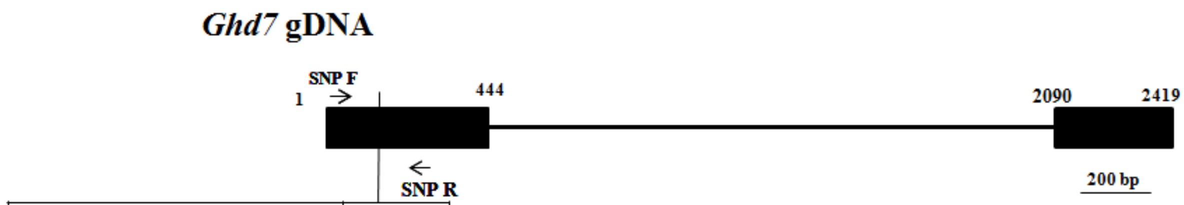

Nucleotide position (bp)	157
Kitaake	T
Dongjin	G
aa substitution	E53*

Figure S2. Schematic diagram of *Ghd7* and GHD7 protein from 'Kitaake' and 'Dongjin'. Arrows indicate primer positions used for genotyping F2 progeny.

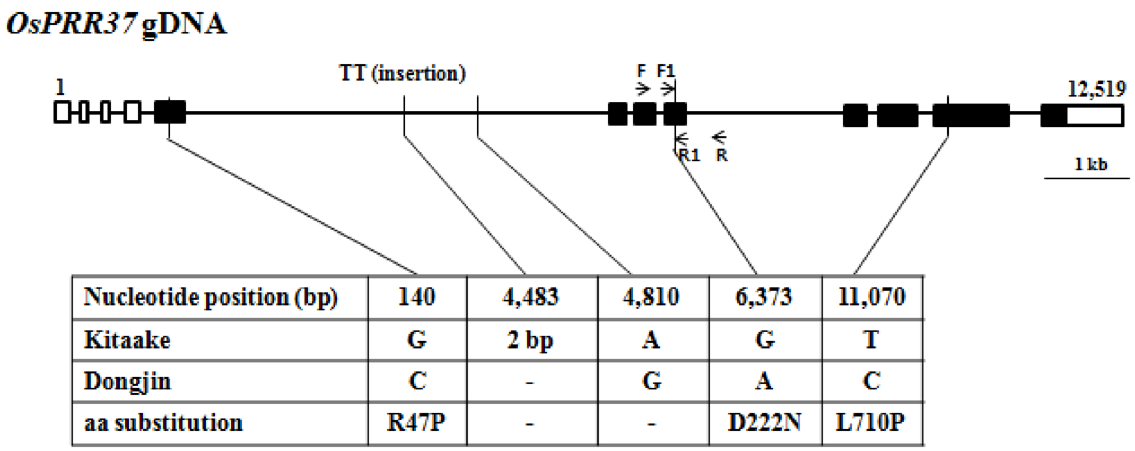

Nucleotide position (bp)	140	4,483	4,810	6,373	11,070
Kitaake	G	2 bp	A	G	T
Dongjin	C	-	G	A	C
aa substitution	R47P	-	-	D222N	L710P

Figure S3. Schematic diagram of *OsPRR37* and its coding protein from 'Kitaake' and 'Dongjin'. Arrows indicate primer positions used for genotyping F2 progeny. F and R1 primers were used for amplifying 'Dongjin' allele; F1 and R primers, for 'Kitaake' allele.

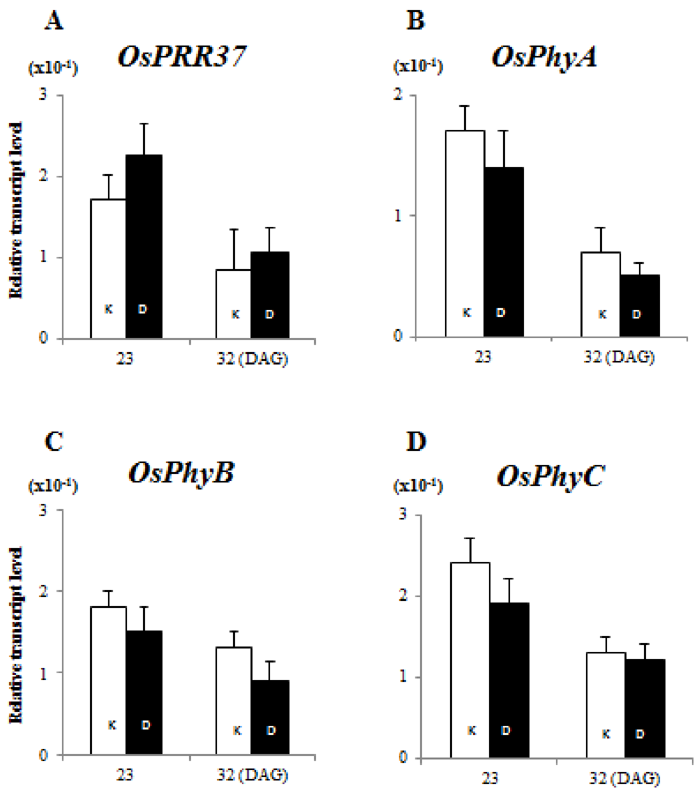

Figure S4. Expression profiles of *OsPRR37*, *OsPhyA*, *OsPhyB*, and *OsPyhC* in ‘Kitaake’ (K) and ‘Dongjin’ (D) at 23 and 32 DAG under LD conditions. RNA was prepared from leaf blades at 2 h after turning on lights. Each data point is average of 2 or more independent experiments. Y-axis, relative values between transcript levels of regulatory gene and *Ubi*; vertical bars, standard deviation; white bar, ‘Kitaake’; black bar, ‘Dongjin’.

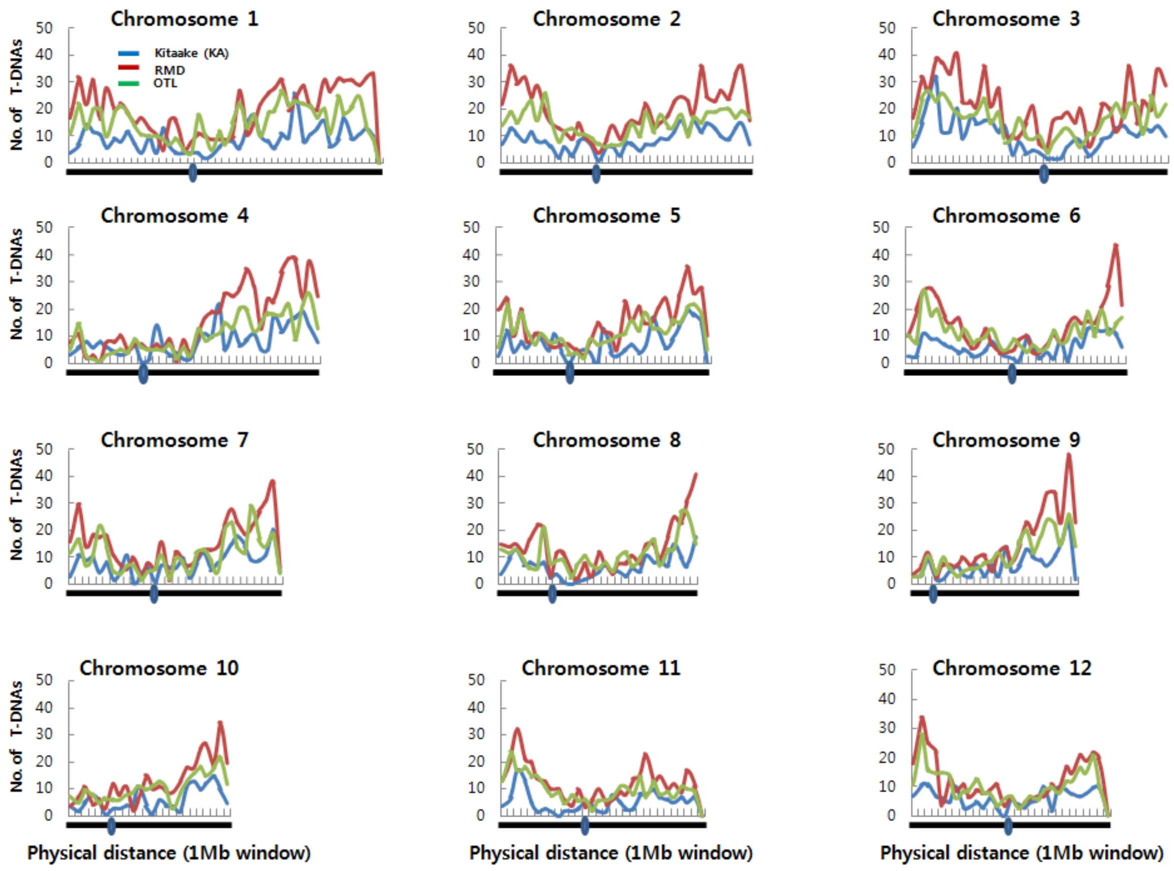

Figure S5. Distribution of T-DNA insertions from ‘Kitaake’, RMD, and OTL along rice chromosomes. Amount of T-DNA from ‘Kitaake’ (blue), RMD (red), and OTL (green) was plotted for each window. Gray circles, centromeric regions.

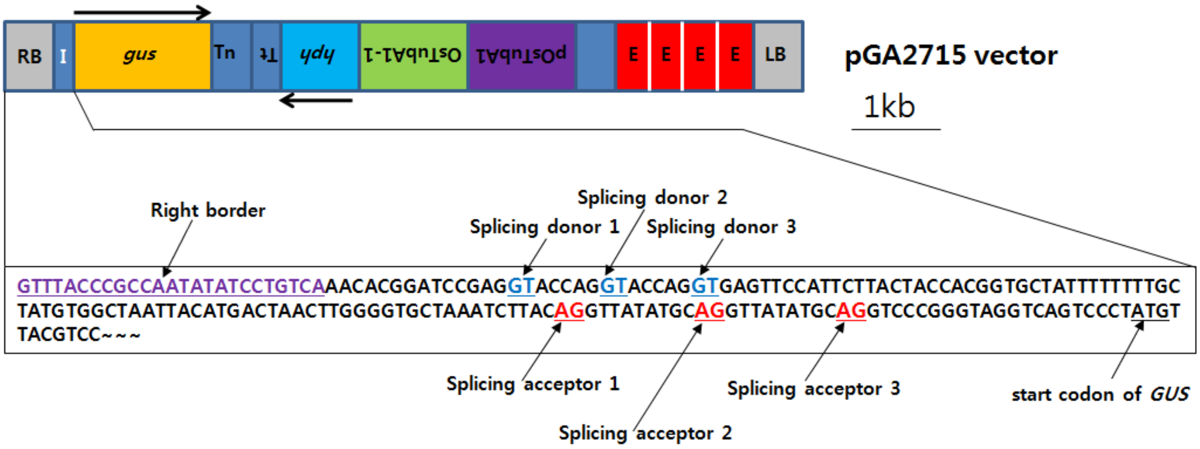

Figure S6. Schematic diagram of T-DNA in pGA2715 vector, and DNA sequence in RB region. RB and LB in gray bars represent right and left borders of T-DNA, respectively. E, enhancer element of CaMV 35S promoter; *hph*, hygromycin phosphotransferase gene; I, *OsTubA1* intron 2 carrying three putative splicing acceptor and donor sites; OsTubA1-1, first intron of *OsTubA1*; pOsTubA1, promoter of *OsTubA1*; Tn, *nos* terminator; Tt, *OsTubA1* terminator.
